# Transcriptomic Analysis in the Hippocampus and Retina of Tg2576 AD Mice Reveals Defective Mitochondrial Oxidative Phosphorylation and Recovery by Tau 12A12mAb Treatment

**DOI:** 10.3390/cells12182254

**Published:** 2023-09-12

**Authors:** Giovanna Morello, Maria Guarnaccia, Valentina La Cognata, Valentina Latina, Pietro Calissano, Giuseppina Amadoro, Sebastiano Cavallaro

**Affiliations:** 1 Institute for Biomedical Research and Innovation, National Research Council (CNR-IRIB), Via Paolo Gaifami, 18, 95126 Catania, Italy; giovanna.morello@irib.cnr.it (G.M.); maria.guarnaccia@cnr.it (M.G.); valentina.lacognata@cnr.it (V.L.C.); 2 European Brain Research Institute (EBRI), Viale Regina Elena 295, 00161 Rome, Italy; v.latina@ebri.it (V.L.); p.calissano@ebri.it (P.C.); giuseppina.amadoro@cnr.it (G.A.); 3 Institute of Translational Pharmacology (IFT), National Research Council (CNR), Via Fosso del Cavaliere 100, 00133 Rome, Italy

**Keywords:** transcriptomic, RNA-seq, GSEA, systems biology, tau immunotherapy, Tg2576, Alzheimer’s disease, mitochondrial bioenergetics, oxidative phosphorylation

## Abstract

Increasing evidence implicates decreased energy metabolism and mitochondrial dysfunctions among the earliest pathogenic events of Alzheimer’s disease (AD). However, the molecular mechanisms underlying bioenergetic dysfunctions in AD remain, to date, largely unknown. In this work, we analyzed transcriptomic changes occurring in the hippocampus and retina of a Tg2576 AD mouse model and wild-type controls, evaluating their functional implications by gene set enrichment analysis. The results revealed that oxidative phosphorylation and mitochondrial-related pathways are significantly down-regulated in both tissues of Tg2576 mice, supporting the role of these processes in the pathogenesis of AD. In addition, we also analyzed transcriptomic changes occurring in Tg2576 mice treated with the 12A12 monoclonal antibody that neutralizes an AD-relevant tau-derived neurotoxic peptide *in vivo*. Our analysis showed that the mitochondrial alterations observed in AD mice were significantly reverted by treatment with 12A12mAb, supporting bioenergetic pathways as key mediators of its *in vivo* neuroprotective and anti-amyloidogenic effects. This study provides, for the first time, a comprehensive characterization of molecular events underlying the disrupted mitochondrial bioenergetics in AD pathology, laying the foundation for the future development of diagnostic and therapeutic tools.

## 1. Introduction

Alzheimer’s disease (AD) is a progressive debilitating neurological condition characterized by memory deficits and cognitive and behavioral impairment. AD incidence, which increases with aging, is the most common and well-known form of dementia in adults, accounting for 60–80% of all cases [1]. Amyloid-positive plaque deposition, tau-laden neurofibrillary tangles and synaptic and neuronal dysfunction are the core neuropathological hallmarks of AD and the disease is preceded by a pre-symptomatic stage that can last for years, during which time the clinical symptoms are undetectable [2]. Despite the amyloid cascade hypothesis dominating the field for years, clinical efforts to target this hallmark have not had robust beneficial effects in preventing or clearly ameliorating AD progression, leading to research exploring other potential mechanisms that could explain or contribute to disease pathogenesis [3,4]. Among others, bioenergetic failure and mitochondrial dysfunction are emerging as two of the factors that may actively contribute to AD onset and progression [5,6,7,8,9].

Mitochondria are intracellular organelles with key roles covering a multitude of metabolic and signaling pathways, including energy metabolism, apoptotic signaling, regulation of innate and adaptive immunity, and the control of second messenger levels, such as calcium and reactive oxygen species (ROS) [5,6]. Neurons critically depend on mitochondrial oxidative phosphorylation to meet energy demands for establishing membrane excitability and executing the complex processes of neurotransmission and plasticity [10]. Therefore, it is not surprising that disturbances in the mitochondrial function in areas highly associated with cognitive function, such as the hippocampus and cortex, may inevitably disturb neuronal function, sensitizing cells to neurotoxic insults and initiating cell death, all significant phenomena associated with the pathophysiology of AD [11,12,13].

A large body of research has shown a consistent and progressive reduction in brain bioenergetics (glucose hypometabolism and mitochondrial dysfunction) in AD patients as well as in multiple preclinical *in vitro* and *in vivo* AD models [14,15,16,17,18,19,20]. In particular, increased aerobic glycolysis, decreased respiratory capacity, increased mitochondrial fragmentation, defective oxidative phosphorylation, Ca^2+^ dyshomeostasis and increased ROS production were reported in the AD context, establishing that these alterations occur early in the course of disease before the onset of significant plaque pathology, suggesting their potential causative role in disease pathogenesis [7,20,21,22,23,24].

In recent years, our research group has extensively evidenced mitochondrial dysfunction not only in hippocampal circuitries but also in the retina of Tg2576 mice—a transgenic model overexpressing a mutant form of APP (isoform 695) with the Swedish mutation (KM670/671NL)—suggesting the intriguing possibility of developing easily measurable retinal biomarkers for early-stage AD diagnosis [25,26,27,28,29,30].

Mounting evidence has also demonstrated that pathological tau directly and/or indirectly impairs mitochondrial structural and functional homeostasis [31,32,33,34]. In this framework, we reported that an NH_2_-derived 20–22 kDa tau fragment (i.e., NH_2_htau) exerts a “gain-of-function” deleterious action into neurons by directly impairing mitochondrial oxidative phosphorylation [25]. Furthermore, we have recently demonstrated that the neuroprotective effect exerted in Tg2576 by the *in vivo* administration of the monoclonal antibody 12A12 (12A12mAb) [35], which specifically neutralizes the neurotoxic NH_2_htau peptide both in the hippocampus and retina, involves, at least in part, metabolic parameters associated with mitochondrial impairment [36].

Despite this growing body of evidence supporting the importance of mitochondrial dysfunction in the pathogenesis of AD, most previous studies were focused on dissecting the alterations of single proteins or genes, leaving the systems biology of the underlying crucial molecular processes largely unexplored. In order to gain additional insights into the molecular mechanisms leading to bioenergetic dysfunctions in AD, here we analyzed transcriptomes of both hippocampal and retinal specimens in parallel from a well-established preclinical AD animal model, Tg2576, and age-matched wild-type (wt) mice, and evaluated the corresponding functional alterations via a Gene Set Enrichment Analysis (GSEA). Compared to traditional single-gene methods based on the identification of differentially expressed genes (DEGs), the GSEA strategy has been extensively validated as a method to identify patterns in gene expression with robust relevance in biological functions that are driven by a large number of genes with moderate but coordinate changes [37,38]. Finally, we also analyzed transcriptomic changes occurring in the hippocampus and retina of Tg2576 mice following treatment with the 12A12mAb tau antibody in order to elucidate the role of mitochondrial-function-associated genes in mediating its beneficial effects *in vivo*. We are confident that the bulk of data obtained with such a parallel approach could give for the first time novel translational insights into the complex genetic syndrome that characterizes AD.

## 2. Materials and Methods

### 2.1. Animals, Ethical Approval and Tissue Collection

All animal procedures and experiments were conducted according to the ARRIVE guidelines and were performed in accordance with the ethical guidelines established by the European Council Directive (Directive 2010/63/EU of 22 September 2010) regarding the care and use of animals for experimental procedures. Experimental approval was obtained from the Italian Ministry of Health (protocol # 1038-2020-PR). This study was carried out according to the 3R principles: Replacement, Reduction and Refinement.

Details regarding animal resources and experimental designs can be found in [36]. Briefly, Tg2576 transgenic mice of both genders (Tg-AD) and their age-matched wt littermates were used at 6 months of age (*n* = 8–10 per group) in the immunization regimen. Tg2576 mice overexpress the human amyloid precursor protein (hAPP), with the Swedish (K670N/M671L) mutation [39], which causes an increase in amyloid β (Aβ) production [40]. Genotyping was carried out to confirm the presence of the human mutant *APP* DNA sequence by PCR. Mice were randomized into three experimental groups: (1) wt mice treated with a saline vehicle; (2) Tg2576 mice treated with a saline vehicle and (3) Tg2576 mice treated with the N-terminal tau 12A12 monoclonal antibody (Tg-AD + mAb; 30 μg/dose). Animals from all experimental groups were sacrificed by cervical dislocation and perfused transcardially with ice-cold phosphate-buffered saline (PBS), their brains and eyes were collected, their hippocampi and retinas were dissected and immediately frozen on dry ice and stored at −80 °C until use.

### 2.2. RNA Extraction and Sequencing

High-quality RNA was extracted from the hippocampi and retinas of wild-type (*n* = 5 hippocampus; *n* = 4 retina), Tg-AD (*n* = 6 hippocampus; *n* = 5 retina) and Tg-AD + mAb (*n* = 6 hippocampus; *n* = 5 retina) mice using Trizol reagent (Sigma-Aldrich, St. Louis, MO, USA) according to the manufacturer’s protocol. The quantity and quality of the extracted RNA were checked by using an Agilent 4200 Tape Station using Agilent High Sensitivity RNA Screen Tape (Agilent Technologies, Santa Clara, CA, USA). Libraries were prepared using a whole transcriptome RNA sequencing methodology, the Ion AmpliSeq Transcriptome Mouse Gene Expression Panel, Chef-Ready Kit on Ion Chef (Thermo Fisher Scientific, Waltham, MA, USA) according to the manufacturer’s instructions. The Ion AmpliSeq Transcriptome Mouse Gene Expression Panel, Chef-Ready Kit allows simultaneous gene expression measurements of >90% representation of mouse RefSeq genes (20,767 well-annotated RefSeq genes + 3163 coding and noncoding transcripts with provisional annotations). Briefly, 10 ng of total RNA was reverse transcribed using a SuperScript VILO cDNA Synthesis Kit (Thermo Fisher Scientific, Waltham, MA, USA) before library preparation via the Ion Chef™ instrument (Thermo Fisher Scientific, Waltham, MA, USA). The resulting cDNA was amplified to prepare barcoded libraries using the Ion Code PCR Plate and the Ion AmpliSeq Transcriptome Mouse Gene Expression Core Panel and Chef-Ready Kit according to the manufacturer’s instructions. Barcoded libraries were quantified using the Ion Library TaqManTM Quantitation Kit (Thermo Fisher Scientific, Waltham, MA, USA) and were combined to a final concentration of 100 pM and used to prepare template-positive Ion Sphere Particles to load on Ion 540 Chips using the Ion 540 Kit-Chef. Sequencing was performed on an Ion S5 sequencer with Torrent Suite Software v6 (Thermo Fisher Scientific, Waltham, MA, USA). Complete high throughput sequencing (RNA-seq) data were deposited to NCBI’s Gene Expression Omnibus (GEO) with the accession number GSE223593.

### 2.3. Transcriptomic Data Analysis

Sequencing data were mapped and analyzed by the Torrent Suite Software version 5.4.0 and the AmpliSeq RNA plugin (Thermo Fisher Scientific, Waltham, MA, USA). Normalized counts (reads per million) were evaluated by the Transcriptome Analysis Console Software (version 4.0.2; Thermo Fisher Scientific, Waltham, MA, USA).

Gene Set Enrichment Analysis (GSEA) was used for functional interpretation of transcriptomic data. This computational method is particularly useful in identifying changes in biological functions that are driven by a large number of genes with moderate changes in comparison to a single-gene analysis. Enrichment analysis was performed using GSEA software (v. 4.2.3) and the Molecular Signatures Database (MSigDB, v2022.1, https://www.gsea-msigdb.org/gsea/msigdb, accessed on 20 April 2023) collection of annotated gene sets from various online pathway databases, gene ontology, gene sets, the biomedical literature and contributions from domain experts [41]. Mouse gene symbols were collapsed to Human Genome Organization (HUGO) symbols with the GSEA tool. GSEA was performed by using normalized expression values and the following default parameters: 1000 gene set permutations, weighted enrichment statistics, gene set size between 15 and 500 and the Signal2Noise metric for ranking genes. The false discovery rate (FDR) was estimated to control the false positive finding of a given normalized enrichment score (NES) by comparing the tails of the observed and null distributions derived from. The NES score indicated whether the genes were mostly up- or down-regulated in a given gene set. A leading-edge analysis was used to identify the core set genes that chiefly account for the enrichment signal [38].

### 2.4. Validation by Reverse Transcription-Quantitative Real-Time Polymerase Chain Reaction (RT-qPCR)

Quantitative real-time PCR (qRT-PCR) was used to validate the gene expression results obtained from the RNA-seq analysis. Seven representative *core set* genes, including *Atp6v1h*, *Eci1*, *Hspa*, *Mrpl15*, *Ndufa9*, *Ndufb2 and Rhot1*, were selected for RT-qPCR validation. Primers used in this study were designed using the Primer-BLAST design tool of the NCBI database (https://www.ncbi.nlm.nih.gov/tools/primer-blast, accessed on 30 May 2023). About 500 ng of total RNA from samples obtained as described above were retrotranscribed to cDNA using a QuantiTect Reverse Transcription kit (Qiagen, Hilden, Germany). Real-time PCR amplification was performed in triplicate using an AriaMx Real-Time PCR System (Agilent Technologies, Santa Clara, CA, USA) by using the Brilliant III Ultra-Fast SYBR^®^ Green qPCR Master Mix (Agilent Technologies, Santa Clara, CA, USA), according to manual’s instructions. Expression levels were normalized by using the β-actin (*Actb*) gene as an internal control. The relative expression was calculated by the 2^−∆∆Ct^ method. Used primers are displayed in Supplementary Table S1.

## 3. Results

### 3.1. Transcriptomic Analysis of Hippocampus and Retina from Symptomatic Tg2576 AD Mice Revealed Defective Mitochondrial Bioenergetics

To gain a mechanistic understanding of bioenergetic dysfunctions in AD from a systems biology perspective, we performed RNA sequencing on hippocampal and retinal specimens from Tg2576 mice at 6 months of age compared with their wt littermates, followed by GSEA analysis to functionally interpret gene expression data. Sequencing metrics for each sample (such as depth of coverage and on-target rate) are reported in Supplementary Table S2.

**Figure 1:**
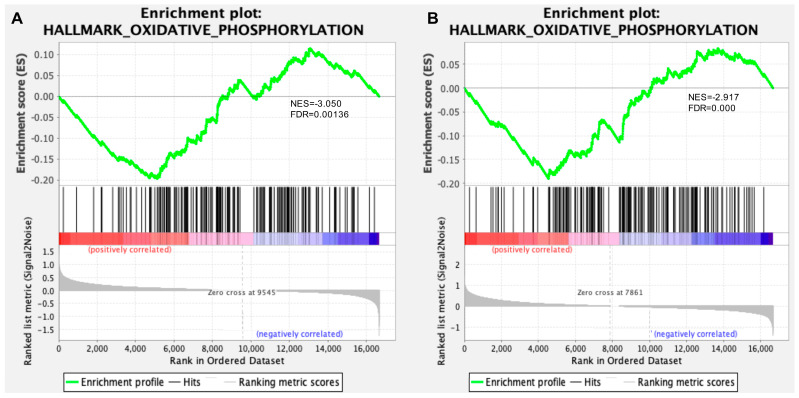
Gene Set Enrichment Analysis (GSEA) supports defective mitochondrial oxidative phosphorylation as a key pathological hallmark of AD. GSEA plots showing enrichment of the “hallmark_oxidative_phosphorylation” gene set in the hippocampus (**A**) and retina (**B**) of in comparison to wt controls. The top portion of each GSEA plot shows the running enrichment score for validated genes as it moves down the ranked list. The bottom portion of each plot shows the value of ranking matrices as it moves down the list of ranked genes. The colored horizontal bar indicates a shift from positively correlated genes (red) to negatively correlated genes (blue). The normalized enrichment scores (NES) and false discovery rate q value (FDR q) are indicated. Further details on GSEA plots can be found at Broad Institute web site (http://www.broadinstitute.org/gsea/index.jsp, accessed on 20 April 2023).

Compared to a DEG-based functional analysis that depends heavily on an arbitrary cutoff to identify significant genes, with a consequent possible underestimation of the biological importance of genes outside of the DEG selection criteria, the GSEA approach uses all of the information about the genes in an experiment to investigate the underlying biological mechanisms. As expected, GSEA results revealed the “hallmark_oxidative_phosphorylation” gene set as the most statistically significant down-regulated gene set in both the hippocampus and retina of Tg2576 AD mice in comparison to their wt littermates (Figure 1, Supplementary Tables S3 and S4). This gene set included genes involved in the regulation of mitochondrial transport and membrane permeability, as well as in various energy metabolism pathways, including lipid oxidation, pyruvate metabolism, mitochondrial electron transport chain and the ATP metabolic process.

**Figure 2:**
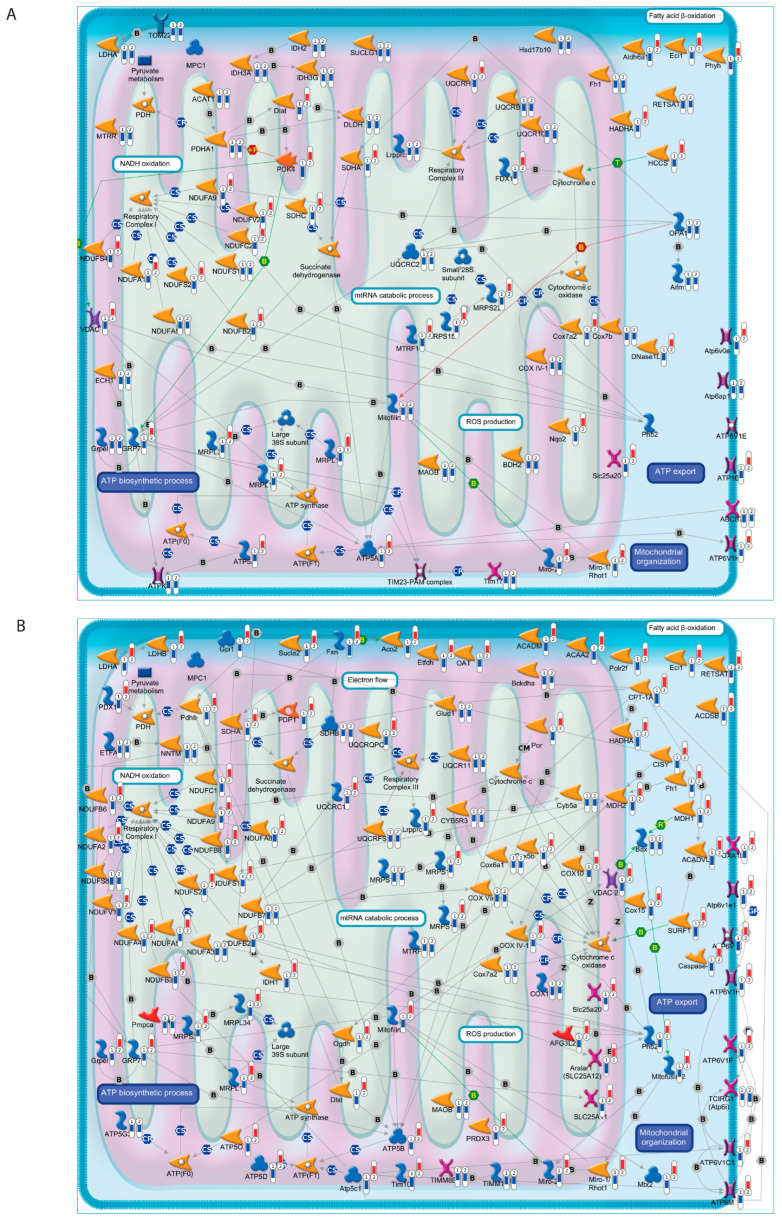
Mitochondrial-related core set genes down-regulated in Tg2576 AD mice contribute to 12A12mAb-mediated neuroprotection. Representative functional illustrations of core set genes that chiefly contribute to the down-regulation of the “*hallmark_oxidative_phosphorylation*” gene set in the (**A**) hippocampus and (**B**) retina of Tg2576 AD mice in comparison to wt controls. The pathway map was created using MetaCore Pathway Map Creator tool (GeneGo). Expression levels (fold change) of *core set* genes in Tg2576 mice vs. wt (thermometer #1) and Tg2576 + mAb vs. Tg2576 mice (thermometer #2) are presented on the map as ‘thermometer-like’ figures (red for up-regulation, blue for down-regulation) and visualized as heatmaps in Supplementary Figures S1 and S2. Further explanations are provided at https://portal.genego.com/legends/MetaCoreQuickReferenceGuide.pdf, accessed on 2 May 2023.

A leading-edge subset analysis was then performed to identify the *core set* of genes that accounts for the gene set enrichment signal. Of the 176 genes associated with oxidative phosphorylation/mitochondrial function, 69 genes were down-regulated in the hippocampus and 106 were down-regulated in the retina of Tg2576 AD in comparison with wt counterparts (Figure 2, Supplementary Figures S1 and S2). Of note, a group of 25 genes were commonly down-regulated between the two tissues and included regulators of the mitochondrial organization and transport (i.e., *Rhot1*, *Rhot2*, *Grpel1*, *Slc25a20*, *Immt*), fatty acid β-oxidation and pyruvate/lactate metabolism (i.e., *Eci1*, *Ldha*, *Sdha*, *Lrpprc*, *Hadha*) and the electron transport chain and ATP metabolic process (i.e., *Cox4i1*, *Cox7a2*, *Ndufs1*, *Ndufs2*, *Ndufa5*, *Ndufb2*, *Ndufa9*) (Figure 2).

### 3.2. Defective Mitochondrial Bioenergetics Are Recovered by 12A12mAb-Mediated Neuroprotection in Both the Hippocampus and Retina of the AD Mouse Model

We have previously demonstrated that *in vivo* administration of the monoclonal antibody 12A12 (12A12mAb), which specifically neutralizes the neurotoxic NH_2_htau peptide, relieves behavioral and neuropathological hallmarks associated with animals’ AD phenotype [42] and normalizes the basal level of L-lactate [36], which may accumulate along the glycolytic route in response to mitochondrial impairment. To investigate the effects of 12A12mAb on mitochondrial bioenergetics, we performed RNA sequencing followed by GSEA analysis on hippocampal and retinal specimens from Tg2576 mice at 6 months of age after immunization with 12A12mAb and compared these with their not-immunized counterpart and wt littermates.

In line with the toxic action of the NH_2_htau peptide on mitochondria functions, we found that the expression levels of the majority of *core set* genes associated with oxidative phosphorylation/mitochondrial function genes (37/69 in hippocampus and 81/106 in retina) were markedly reverted in the Tg2576 AD mouse model following 12A12mAb treatment (Figure 2 and Supplementary Figure S1). Within this context, interesting examples are the coordinated down-regulated expression of a large number of genes that encode the mitochondrial respiratory chain complexes I (*Ndufa1*, *Ndufa2*, *Ndufa3*, *Ndufa5*, *Ndufa8*, *Ndufa9*, *Ndufb2*, *Ndufb3*, *Ndufb7*, *Ndufc2*, *Ndufs1*, *Ndufs2*, *Ndufs3*, *Ndufs4*, *Ndufs8*, *Ndufs7*, *Ndufv1*, *Ndufv2*), II (*Sdha*, *Sdhb*, *Sdhc*), III (*Uqcrc1*, *Uqcrc2*, *Uqcr10*, *Uqcr11*, *Uqcrfs1*, *Uqcrb*, *Uqcrh*, *Uqcrq*), IV (*Cox4i1*, *Cox5a*, *Cox5b*, *Cox6a1*, *Cox7a2*, *Cox7b*, *Cox10*, *Cox11*, *Cox15*) and V (*Atp5a1*, *Atp5b*, *Atp1b1*, *Atp5c1*, *Atp5d*, *Atp5g3*, *Atp5j*, *Atp5j2*, *Atp5o*) in the hippocampus and/or retina of Tg2576 AD mice whose alterations were recovered following *in vivo* administration of 12A12mAb (Figure 2, Supplementary Figures S1 and S2). Several genes encoding proteins involved in the maintenance of mitochondrial membrane potential (*Atp6v0e*, *Atp6v1e1*, *Atp6v1c1*, *Atp6v1d*, *Atp6v1f*, *Atp6v1h*, *Atp6v1h*, *Atp6ap1*, *Oxa1l*, *Slc25a3*, *Slc25a12*, *Slc25a20*, *Slc25a20*, *Slc25a11*, *Timm8b*, *Timm10*, *Timm17a*, *Tomm22*, *Timm13*, *Tcirg1*), as well as in the mtRNA stabilizing protein (*Lrpprc*) and mitochondrial redox state (*Nnt*), were also down-regulated in Tg2576 mice and their expression was strongly rescued by 12A12mAb immunization (Figure 2, Supplementary Figures S1 and S2). A group of seven *core set* genes (*Atp6v1h*, *Eci1*, *Hspa*, *Mrpl15*, *Ndufa9*, *Ndufb2*, *Rhot1*), showing an increased expression following 12A12mAb treatment in both the retina and hippocampus, were selected for RNA-seq transcription validation using qRT-PCR (Supplementary Figure S3). The relative expression patterns of the tested *core set* genes were found to be positively correlated with the fold change variations obtained from the RNA-seq results (Supplementary Figure S4).

## 4. Discussion

Although multiple lines of evidence implicate energy deficiency and mitochondrial dysfunction as early pathomechanisms in AD, the molecular mechanisms leading to mitochondrial failure are still to be further investigated and understood [6,7,8,9,19,20,21,23,26,27,29,31,32,33,34,43]. The purpose of this study was to provide a systems biology characterization of the molecular mechanisms underlying bioenergetic dysfunctions in AD. To address this, we analyzed transcriptomes (RNA-seq data) of the hippocampus and retina of Tg2576 AD mice in comparison with their wt littermates, assessing the functional relevance of their gene expression changes in the context of mitochondrial bioenergetic processes.

In line with findings that emerged from our previous work [36], our transcriptomic analysis revealed a comprehensive decrease in the expression of genes involved in mitochondrial/oxidative phosphorylation pathways both in the hippocampus and retina of Tg2576 AD mice (Figure 1), further sustaining the essential role of mitochondria bioenergetic dysfunction in the pathogenesis of AD and supporting its potential utility as a disease biomarker.

Among the *core set* genes of the mitochondrial oxidative phosphorylation pathway, we observed the coordinated downregulation of genes encoding components of mitochondrial pyruvate metabolism/catabolism, such as pyruvate dehydrogenase complex (*Pdha1*, *Pdhb*, *Pdk4*, *Pdp1*, *Pdhx*, *Dlat*, *Dldh*, *Dlst*, *Acat1*), succinate dehydrogenase (*Sdha*, *Sdhb*, *Sdhc*), lactate dehydrogenase (*Ldha*, *Ldhb*) and the mitochondrial fatty acid β-oxidation pathway (*Aldh6a1*, *Eci1*, *Hsd17b10*, *Hadha*, *Retsat*, *Acadsb*, *Acadm*, *Acadvl*, *Acca2*, *Aco2*, *Cpt1a*, *Oat*, *Etfdh*, *Bckdha*) (Figure 2). Significant reductions in the expression and activity of pyruvate dehydrogenase complex and lactate dehydrogenase were previously reported in *post mortem* brain tissues from Alzheimer’s patients, as well as in animal models of AD, demonstrating how the accumulation of lactate due to these metabolic defects may impair neuronal bioenergetics and result in cognitive decline and neurodegeneration [7,44,45,46,47,48,49,50,51,52,53]. In this regard, our previous functional and metabolic findings highlight a general decline in glucose utilization and elevated levels of L-lactate both in hippocampal and, even more, retinal homogenates from Tg2576 AD mice in comparison with their wild-type littermates [36].

A decreased expression of several components of the mitochondrial respiratory chain complexes I (*Ndufa1*, *Ndufa2*, *Ndufa3*, *Ndufa5*, *Ndufa8*, *Ndufa9*, *Ndufb2*, *Ndufb3*, *Ndufb7*, *Ndufc2*, *Ndufs1*, *Ndufs2*, *Ndufs3*, *Ndufs4*, *Ndufs8*, *Ndufs7*, *Ndufv1*, *Ndufv2*), II (*Sdha*, *Sdhb*, *Sdhc*), III (*Uqcrc1*, *Uqcrc2*, *Uqcr10*, *Uqcr11*, *Uqcrfs1*, *Uqcrb*, *Uqcrh*, *Uqcrq*), IV (*Cox4i1*, *Cox5a*, *Cox5b*, *Cox6a1*, *Cox7a2*, *Cox7b*, *Cox10*, *Cox11*, *Cox15*) and V (*Atp5a1*, *Atp5b*, *Atp1b1*, *Atp5c1*, *Atp5d*, *Atp5g3*, *Atp5j*, *Atp5j2*, *Atp5o*) was also found in the hippocampus and/or retina of Tg2576 AD mice. Differential expression of these mitochondrial complexes has been previously reported in brain and peripheral tissues of AD patients and animal models, and their functional defects were proposed as a driver of AD pathology by determining a decreased cellular energy production, an increased ROS level and a depolarized mitochondrial membrane, which in turn cause a dramatic effect on ATP production and protein transport across mitochondrial membranes [54,55,56,57,58,59,60]. Dysregulation of mitochondrial redox states in AD also emerged by the decreased expression of nicotinamide nucleotide transhydrogenase (*Nnt*) in the retina of Tg2576 mice (Figure 2). *Nnt* encodes a key anti-oxidative enzyme that regenerates NADPH from NADH, and mutations or deletions in this gene impair mitochondrial function, causing a shift to an oxidized redox state that is deleterious in AD [61,62].

As previously demonstrated, Aβ deposition promotes dysfunction of mitochondria also by altering membrane permeability, reducing membrane potential and mtRNA stability and increasing ROS generation, eventually leading to nerve cell death [63,64,65,66]. In agreement with these findings, we observed a reduced expression in Tg2576 mice of several genes encoding proteins involved in the maintenance of mitochondrial membrane potential (*Atp6v0e*, *Atp6v1e1*, *Atp6v1c1*, *Atp6v1d*, *Atp6v1f*, *Atp6v1h*, *Atp6v1h*, *Atp6ap1*, *Oxa1l*, *Slc25a3*, *Slc25a12*, *Slc25a20*, *Slc25a20*, *Slc25a11*, *Timm8b*, *Timm10*, *Timm17a*, *Tomm22*, *Timm13*, *Tcirg1*), as well as in mitochondrial transport and organization (*Lrpprc*, *Rhot1*, *Rhot2*) (Figure 2). Consistent with our findings, deficiency of the vacuolar (H+) ATPase (V-ATPase), a large multi-subunit complex of ATP-driven proton pumps, was detected in AD patients and animal models and associated with defective cognitive functions by triggering defects in oxidative phosphorylation, autophagy and axonal transport and synaptic transmission alterations [65,67,68,69,70,71,72,73]. Although the role of the SLC25 family of mitochondrial transporters in the brain is fairly unknown, a reduced expression of some of its members was detected in the brain of AD patients and associated with tau hyperphosphorylation and Aβ deposition [74,75,76]. A significant reduction in *LRPPRC*, a known mtRNA-stabilizing protein, was found in AD brain samples, while its knockdown causes a general decrease in mitochondrial mRNA levels, impaired translation and a general decrease in respiratory chain complexes [77,78]. The outer mitochondrial membrane proteins Miro1/2 (encoded by the *Rhot1* and *Rhot* genes, respectively) play a vital role in the maintenance of mitochondrial dynamics, calcium homeostasis, axonal transport and cellular energy generation, and their involvement in the pathogenesis of AD has recently been proposed [79,80,81,82].

With the aim to validate our results, we applied a GSEA analysis to an independent data set deposited in GEO (accession number: GSE31624) that includes gene expression profiles in the hippocampus of 5-month-old heterozygous Tg2576 mice and wt littermates. This analysis confirmed “oxidative phosphorylation” as the most down-regulated gene set in the hippocampus of early symptomatic Tg2576 mice, further supporting the relevance of this mechanism in AD pathology (Supplementary Table S5). In addition, we also compared our gene expression data with those detected in the cerebral cortex of Tg2576 mice long before (2 months old), immediately before (5 months old) and after (18 months old) the appearance of amyloid pathology and cognitive impairment in order to establish whether emerging mitochondrial dysfunction can be detected early in the course of AD and represent a valid disease biomarker [24]. To this end, our comparative analysis identified some of our *core set* genes (*Atp6ap1*, *Atp6v1c1*, *Cox4i1*, *Cox7a2*, *Cs*, *Gpi1*, *Sucla2*, *Uqcrc1*) were significantly down-regulated in the cerebral cortex of Tg2576 mice during the earliest pre-symptomatic stages, supporting their potential role in early-stage AD diagnosis [24].

Besides providing new insights into the importance of mitochondrial injury in the etiology and pathogenesis of AD, our results also offer interesting clues on the possibility of targeting these mitochondrial defects to counteract neurodegeneration in AD. In our previous work [36], we showed that the neuroprotective effect exerted in Tg2576 by the *in vivo* administration of 12A12mAb, a monoclonal antibody that neutralizes the neurotoxic NH_2_htau peptide, is associated with the mitigation of mitochondrial energetic dyshomeostasis, both in the hippocampus and retina. In line with these results, we found that the expression levels of the majority of *core set* genes (retina: 76%; hippocampus: 53%) were reverted to their normal level after treatment with the 12A12mAb tau antibody (Figure 2 and Supplementary Figure S1), further supporting mitochondrial metabolism as a key mechanism involved in 12A12mAb-mediated neuroprotection. Concerning the mechanistic link between the administration of 12A12mAb targeting the NH_2_htau peptide and the transcriptomic changes in the gene expression of mitochondrial function, it is noteworthy that a sizeable amount of tau, under physiological conditions, binds chromatin and nucleic acids. However, it seems that pathological (hyperphosphorylated and/or truncated) tau species accumulate in the nucleus of neurons, locally exerting a harmful effect that further accelerates neurodegeneration [83,84].

## 5. Conclusions

Our findings, for the first time, offer a systems biology perspective of disrupted mitochondrial bioenergetics in AD pathology and its recovery following 12A12mAb treatment, highlighting its potential utility for the development of useful early biomarkers and future disease-modifying therapies for AD.

## Data Availability

The data that support the findings of this study have been submitted to the NCBI Gene Expression Omnibus (GEO) with the accession number GSE223593.

## Supplementary Materials

The following supporting information can be downloaded at: https://www.mdpi.com/article/10.3390/cells12182254/s1. Figure S1. Heat maps showing the expression levels (fold change) of the core set genes in both the hippocampus and retina under various experimental comparisons. Figure S2. Heatmaps showing the expression levels (log2 of counts normalized by RPM) of *core set* genes in the hippocampus and retina of each sample. Table S1. Primers used for real-time RT-PCR. Figure S3. Validation of RNA sequencing by RT-qPCR. Figure S4. Comparison of RNA-Seq analysis with RT-qPCR validation assays. Table S2. Sequencing coverage metrics for each sample. Table S3. Gene set enrichment analysis (GSEA) results of transcriptome data in the hippocampus and retina of Tg2576 vs. wt mice. Table S4. Detailed results of the leading-edge set analysis in GSEA for the enriched gene set “OXIDATIVE_PHOSPHORYLATION” in the hippocampus and retina of Tg2576 vs. wt mice. Table S5. Gene set enrichment analysis (GSEA) results of transcriptome data in the hippocampus of 5-month-old heterozygous Tg2576 mice and wt littermates (GSE31624).
