# Supplementary figures and images for: Transcriptomic Analysis in the Hippocampus and Retina of Tg2576 AD Mice Reveals Defective Mitochondrial Oxidative Phosphorylation and Recovery by Tau 12A12mAb Treatment
Source: Cells. 2023 Sep 12;12(18):2254. doi: 10.3390/cells12182254 (PMC10527038; doi:10.3390/cells12182254)

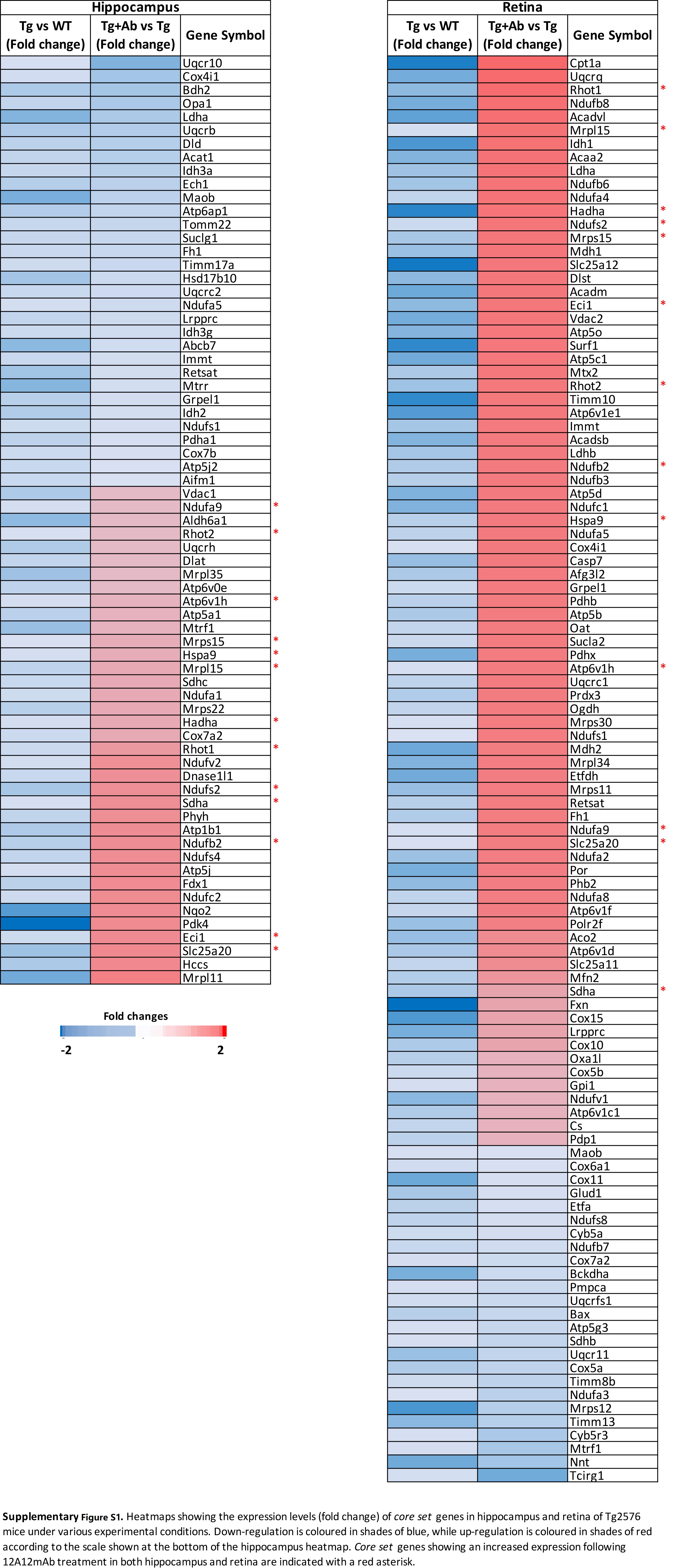

Supplement: Supplementary file 1 [file cells-12-02254-s001.zip › Supplementary Figure S1.jpg]

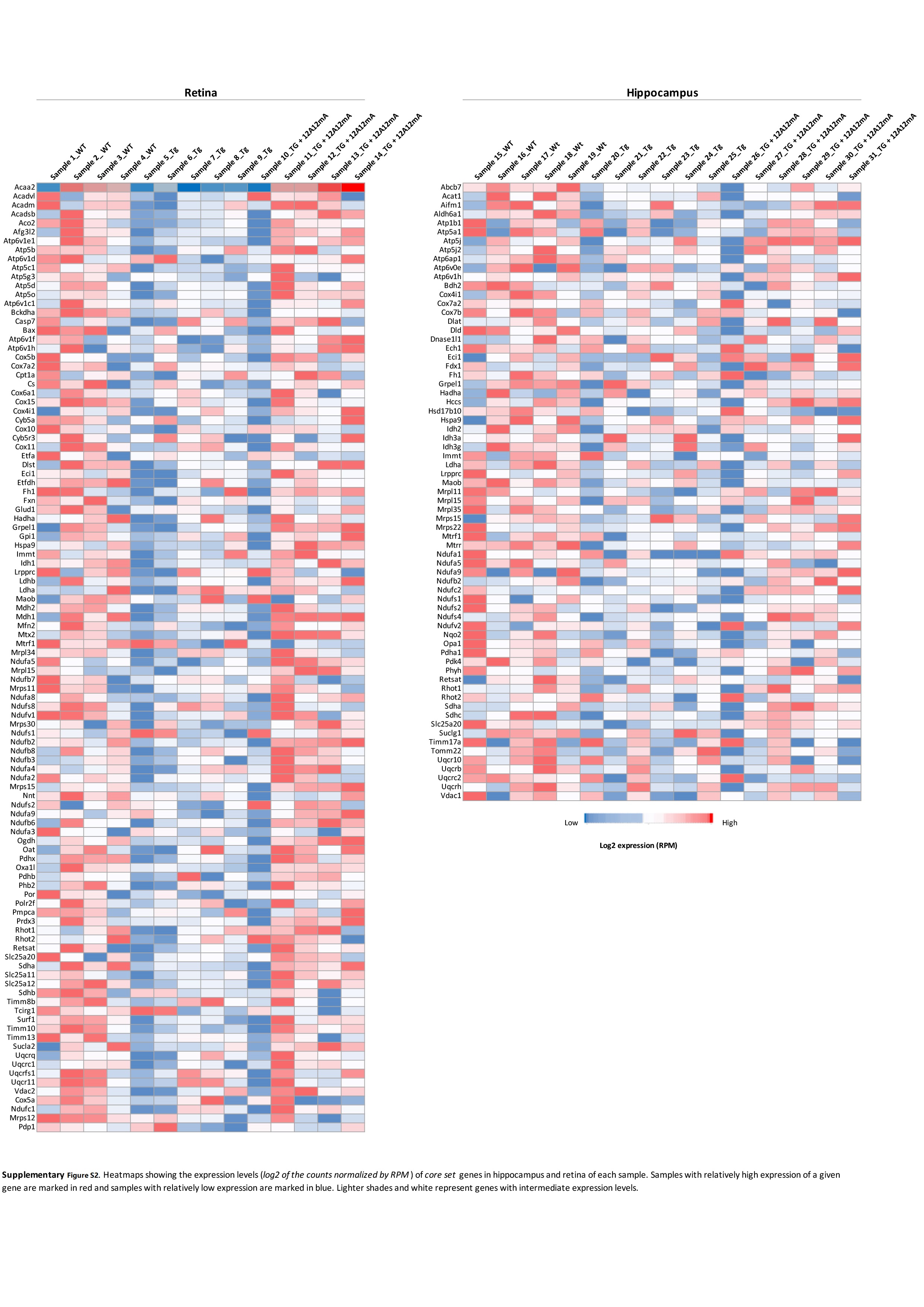

Supplement: Supplementary file 1 [file cells-12-02254-s001.zip › Supplementary Figure S2.jpg]

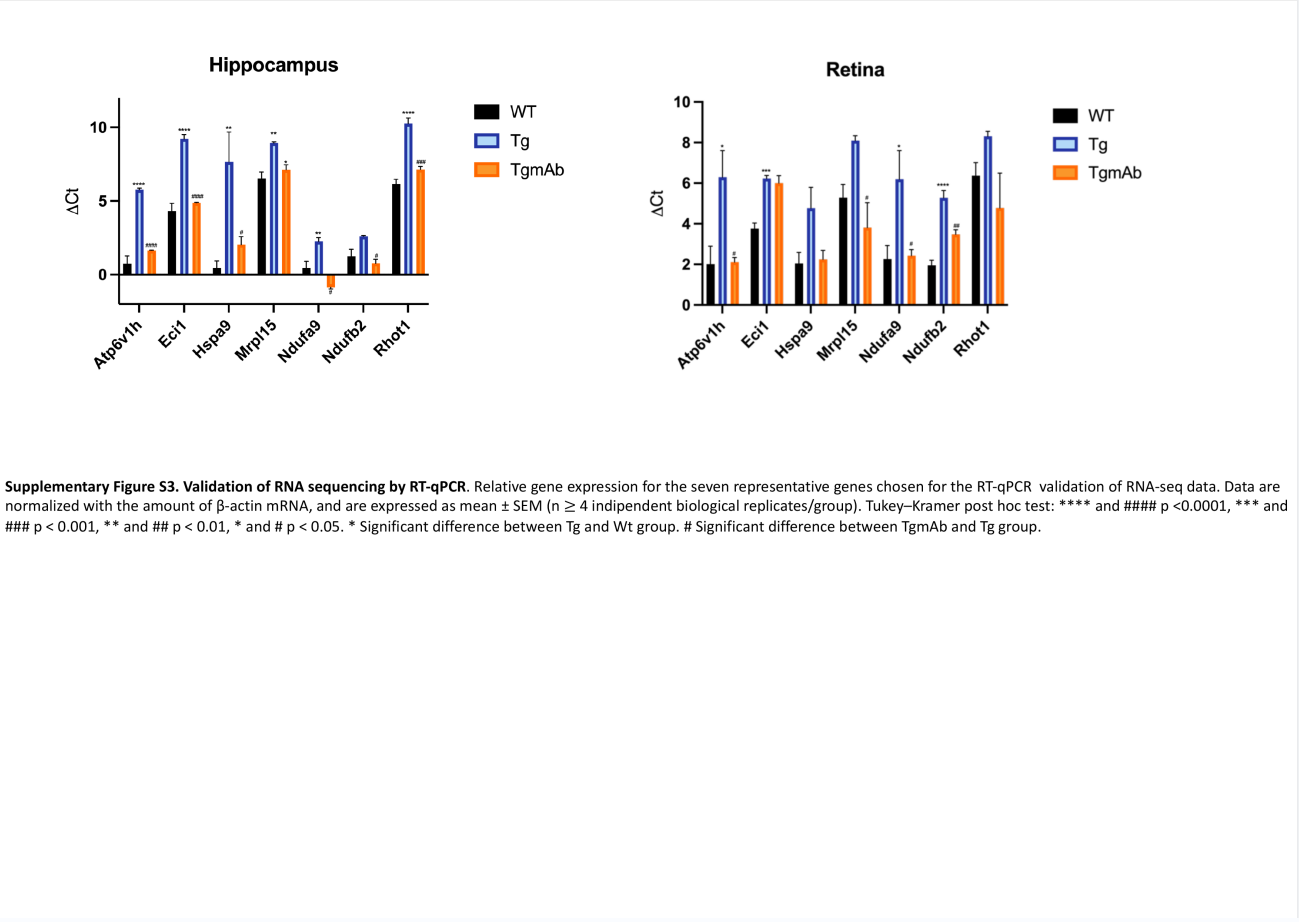

Supplement: Supplementary file 1 [file cells-12-02254-s001.zip › Supplementary Figure S3.png]

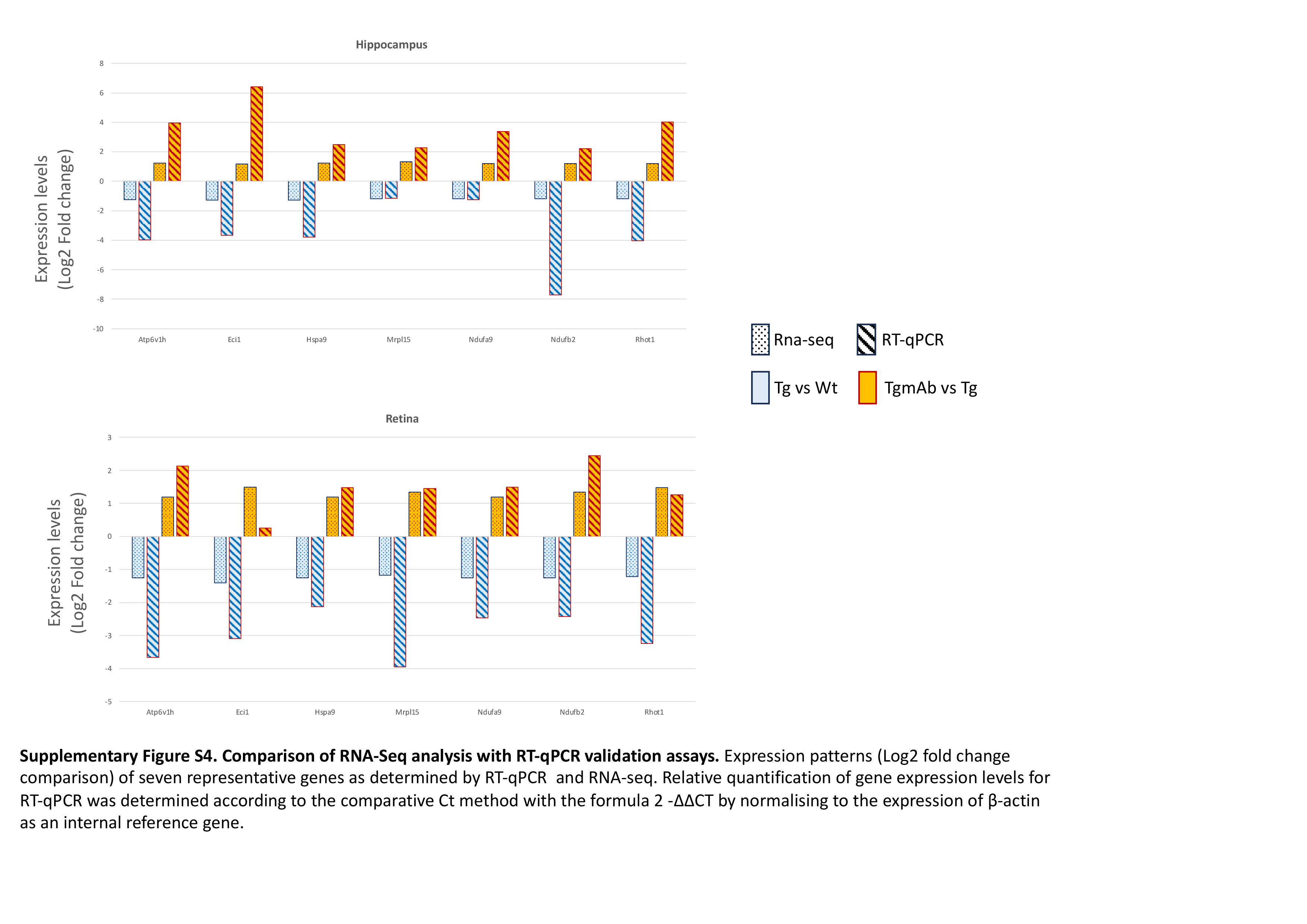

Supplement: Supplementary file 1 [file cells-12-02254-s001.zip › Supplementary Figure S4.jpg]
